# Scene semantics affects allocentric spatial coding for action in naturalistic (virtual) environments

**DOI:** 10.1038/s41598-024-66428-9

**Published:** 2024-07-05

**Authors:** Bianca R. Baltaretu, Immo Schuetz, Melissa L.-H. Võ, Katja Fiehler

**Affiliations:** 1 Department of Experimental Psychology, Justus Liebig University Giessen, Otto-Behaghel-Strasse 10F, 35394 Giessen, Hesse Germany; 2 Department of Psychology, Goethe University Frankfurt, 60323 Frankfurt am Main, Hesse Germany

**Keywords:** Spatial coding, Scene semantics, Scene perception, Memory-guided action, Virtual reality, Cognitive neuroscience, Sensory processing, Object vision

## Abstract

Interacting with objects in our environment requires determining their locations, often with respect to surrounding objects (i.e., allocentrically). According to the scene grammar framework, these usually small, *local* objects are movable within a scene and represent the lowest level of a scene’s hierarchy. How do higher hierarchical levels of scene grammar influence allocentric coding for memory-guided actions? Here, we focused on the effect of large, immovable objects (*anchors)* on the encoding of local object positions. In a virtual reality study, participants (n = 30) viewed one of four possible scenes (two kitchens or two bathrooms), with two anchors connected by a shelf, onto which were presented three local objects (congruent with one anchor) (Encoding). The scene was re-presented (Test) with 1) local objects missing and 2) one of the anchors shifted (Shift) or not (No shift). Participants, then, saw a floating local object (target), which they grabbed and placed back on the shelf in its remembered position (Response). Eye-tracking data revealed that both local objects and anchors were fixated, with preference for local objects. Additionally, anchors guided allocentric coding of local objects, despite being task-irrelevant. Overall, anchors implicitly influence spatial coding of local object locations for memory-guided actions within naturalistic (virtual) environments.

## Introduction

In our everyday interactions with the objects around us, we form goal-directed movement plans toward specific targets. When we formulate a particular goal, we must create relationships between ourselves (our specific effector of choice, such as the eye or hand) and the desired object with which we wish to interact (e.g., a racquet). Doing this requires specific calculations that can be performed using one of two possible frames of reference. The first involves using an *egocentric* coding scheme, whereby a target object is related to the self^1–3^. This is often the reference frame that is tapped into when visual information about the target is presently available. However, under circumstances where visual signals start to decay over time, such as for memory-guided actions, another, *allocentric* reference frame allows the tapping into of object-to-object spatial relations^4–6^. When we engage memory to perform an action toward a desired object, for example grabbing the cup of tea while getting pulled into an exciting novel, we rely more on the latter to help us remember the location of the object that we want to reach toward while keeping our eyes fixed at another location^7^.

Is allocentric coding a rigid and single-shot process or is it susceptible to and influenced by low- to high-level cognitive factors? Previous research has shown that even coloured dot stimuli presented against a simple, monochromatic background can be influenced, in terms of their remembered locations, by the shifting of a landmark to which the target is related^1,4,8,9^. Within these more simplistic scenarios, it has also been shown that stimulus features, such as size and proximity, have the ability to influence the coded object-to-object relations that guide our memory-guided actions^4,8^. The extent to which target and landmark are weighted in their spatial relationship has also been shown to occur in an optimized manner, demonstrated by shifting of surrounding landmarks and noting the degree to which the target is also shifted^10^. While abstract targets and scenes are useful in showing some of the influential factors, especially at the object level, that exist for spatial coding, they are simultaneously limited in helping to extract information about additional, complex cognitive factors that may also play a role. To target these types of contributors, more naturalistic objects, scenes, and set-ups have been implemented^5,11^. With these stimuli, it has been shown that, in addition to low-level factors, prior knowledge^12^, task relevance^13^, and scene coherence^14^ can influence how targets are coded allocentrically for reach. New technologies like virtual reality provide the possibility to study spatial coding in more naturalistic 3D environments (e.g.,^13,15^). For example, Karimpur and colleagues^16^ immersed participants in a virtual environment, where they were asked to encode a table scene with six objects (three fruits and three office objects) and after a short memory delay, reach to indicate the position of the missing object. They found that the remembered target object position was influenced by a shift of the surrounding objects and that this influence was twice as strong when the surrounding objects were from the same semantic object category^16^. However, when we think of objects in various environments, they are rarely encountered in isolation, but are rather embedded in more complex, intricate scenes that might provide additional sources of information that could influence to how we spatially encode objects.

When we are faced with situations or tasks that require searching for or recalling a specific object location in naturalistic (virtual) scenes, it has been shown that there is a hierarchy of scene processing that can influence these goals^17,18^. Scene perception has been shown to be influenced by *semantics* (providing information about *what* objects to expect in a given scene category) and *syntax* (describing the spatial relationships between objects within a scene; see ^19,20^ and ^17,21^). Moreover, scenes tend to be organized according to a ‘grammatical’ hierarchy, with scenes at the top of the hierarchy, which can be further subdivided into so-called “phrases”, which again consist of so-called “*anchors*”, which are typically larger, immovable objects (e.g., a kitchen sink). These are interconnected with the next level of the hierarchy by predicting the identity and location of smaller, movable “local objects” one is likely to find in the anchor’s proximity (e.g., the soap on top of the sink)^17,18,22^ (for reviews, see Võ et al.,^17,18^).

The level of phrases tends to be structured based on the type of actions that can be performed therein (see also “reach spaces” by^17,18,23^). The relationships that exist between anchors and local objects within these phrases are key in efficiently guiding real-world search and also affect memory for the identity and location of objects^17,18,18,22^. Given that local-to-local object interactions for spatial coding are influenced by object semantics^16^, and memory for local objects is under the influence of anchors within scenes^22^, we wanted to investigate whether and how scene-level and phrasal-level semantics influences allocentric coding of local objects for memory-guided actions.

**Figure 1:**
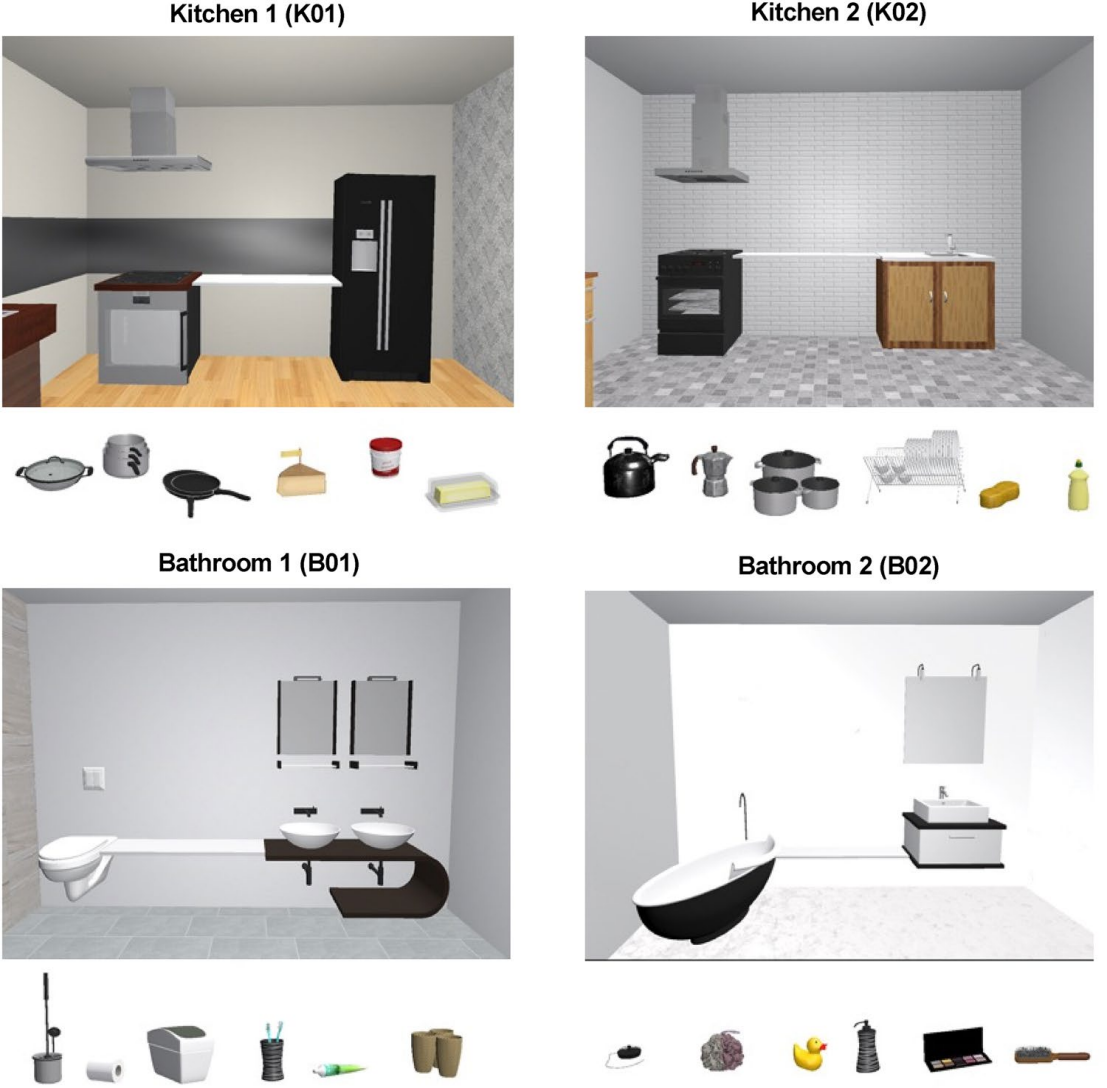
Example virtual scenes for the kitchen and bathroom. To create some variety, we used two example scenes for each of the kitchen and bathroom, given the strong associations formed between local and anchors therein. In each of the scenes shown, there is a shelf that is flanked on either side by an anchor. Local objects are distributed and presented along the shelf at the appropriate time. All local objects and anchors presented in a given scene are congruent with scene-level semantics.

In this experiment, we created naturalistic (virtual) scenes that consisted of anchor objects that were always congruent at the scene level (anchor-scene semantics) and either congruent or incongruent at the phrase level (anchor-local object semantics). Participants viewed one of two possible scene categories (kitchen or bathroom) for a short, limited time. During this Encoding phase, they viewed three local objects presented on a shelf extending between two anchors (Fig. 1). In the Test Phase we implemented two conditions: In a No-shift condition, the anchors were presented in their original positions, whereas in the Shift condition, one of the two anchors was shifted (rightward or leftward) imperceptibly (in the absence of the local objects). In the final, Response phase, one of the three local target objects reappeared, floating in front of the participant, who was asked to then virtually grasp the object with a controller and walk to reposition it on the shelf where they remembered it to have been. We had two hypotheses: (1) Scene-level Semantics: if anchors implicitly influence the spatial coding of local objects in scenes, any anchor shift should result in a difference, or error, of the placement response in the direction of the shift; and (2) Phrasal-level Semantics: if anchors are semantically congruent with the local object target, placement errors should be larger (regardless of direction) compared with incongruent ones. As eye movements are indicative of viuospatial attention and thus can inform about task-relevant areas and objects in the scene^12,13^, we followed an exploratory approach in examining gaze behaviour. In preview of the results, we found that placement errors for local objects were generally influenced by anchors’ shifts (confirming our Scene-level Semantics hypothesis), but not by the semantic anchor-local object relationship (contradicting the Phrasal-level Semantics hypothesis).

## Methods

### Participants

To determine the required number of participants, we performed a power analysis (G*Power v3.1.9.6;^24^) using the following parameters: an effect size of 0.36 based on Karimpur et al.^16^, alpha level of 0.05, two groups, two measurements, and a correlation of 0.49, with a desired minimum power of 0.95. We determined that 28 participants were required and would provide an actual power of 0.95. In order to meet this minimum sample size, given that we used three stimulus arrangements (variations of the left–right order of the presented stimuli), we had 10 participants per arrangement, bringing our total number of participants to 30 (age: 26.2 + / − 4.6; 18 females). No participants had any neurological impairments. All participants had normal or corrected-to-normal vision. Handedness was tested using the Edinburgh Handedness Inventory^25^; score across participants: 89.38 + / − 19.79; all participants, except for one ambidextrous individual, were right-handed). Participants received financial remuneration or participation credits. All participants provided written informed consent. The experimental protocol was in concordance with the general principles of the Declaration of Helsinki (2013; without preregistration of the study) and approved by the local ethics committee (Department of Psychology) at Justus-Liebig-University Giessen (Germany).

### Apparatus

All stimuli were displayed using the HTC Vive Pro Eye VR head-mounted display (HMD) (HTC Corporation, Xindian, New Taipei, Taiwan). The HMD had a resolution of 1440 × 1600 pixels per eye, and a refresh rate of 90 Hz. We used SteamVR (v. 1.20.1) to run the HMD, on a Dell Alienware computer (Intel Core i9 7980XE processor, 32 GB RAM, with two NVIDIA GeForce GTX 1080Ti graphics processors), within Unity (v. 2020.3; Unity Technologies, Inc., San Francisco, CA, USA) together with the Unity Experiment Framework^26^. Eye movements were recorded using the inbuilt eye tracker within the HMD^27,28^. To obtain placement data, we employed the use of a Vive Pro controller (HTC Corporation, Xindian, New Taipei, Taiwan). Four lighthouse base stations (one in each of the four corners of the lab) were used to track the position of the HMD and controllers throughout the experiment.

### Set-up and stimuli

In this experiment, we used two types of scenes (two kitchen and two bathroom scenes), in which we included two major types of objects: (1) anchor and (2) local objects^17^. Each scene was presented always centred relative to the shelf (~ 1 m in length), connecting the two anchors, to avoid skewed viewing of the scene. The anchors (Fig. 1) differed within the categories and specific scenes (kitchen 1: stove and fridge; kitchen 2: stove and sink; bathroom 1: sink and bathtub; bathroom 2: sink and toilet), but were congruent with the scene type (e.g., stove in the kitchen).

Within a given trial, three local objects were presented on the shelf, extending between two anchors. To manipulate the congruency of phrasal-level semantics, the local objects were always (1) semantically congruent with one anchor (e.g., liquid soap and the kitchen sink) and (2) semantically incongruent with the other anchor in the same scene (e.g., liquid soap and the stove in the kitchen). Anchors and local objects were always congruent with regard to scene-level semantics (e.g., sink in the bathroom; milk, butter, and yogurt in the kitchen). Within each of the four scenes, three possible positions were chosen at random along the length and depth of the shelf, such that objects maintained a relative distance between themselves, never allowing for occlusion. Local objects could also never be positioned within less than 0.075 m of the edges of the shelf. To avoid any order effects of the local objects, we created three arrangements by pseudorandomly shuffling the local objects among the three chosen locations on the shelf within each scene.

Scenes and objects therein were produced and provided by the Scene Grammar Lab^29^. Additional local objects were purchased and added from TurboSquid (www.turbosquid.com). The stimuli and scenes were presented within a virtual room (2.5 m × 3 m) that fit within the experimental room.

### Procedure

Each trial commenced with the presentation of an instruction screen and a virtual pair of blue shoes that indicated to participants where they should stand in the virtual room. The positioning of the standing position was aligned with the centre of the shelf, so that participants had equal / central viewing of each scene. Upon finding their position, according to the instructions, they were required to press the controller button to start the trial.

**Figure 2:**
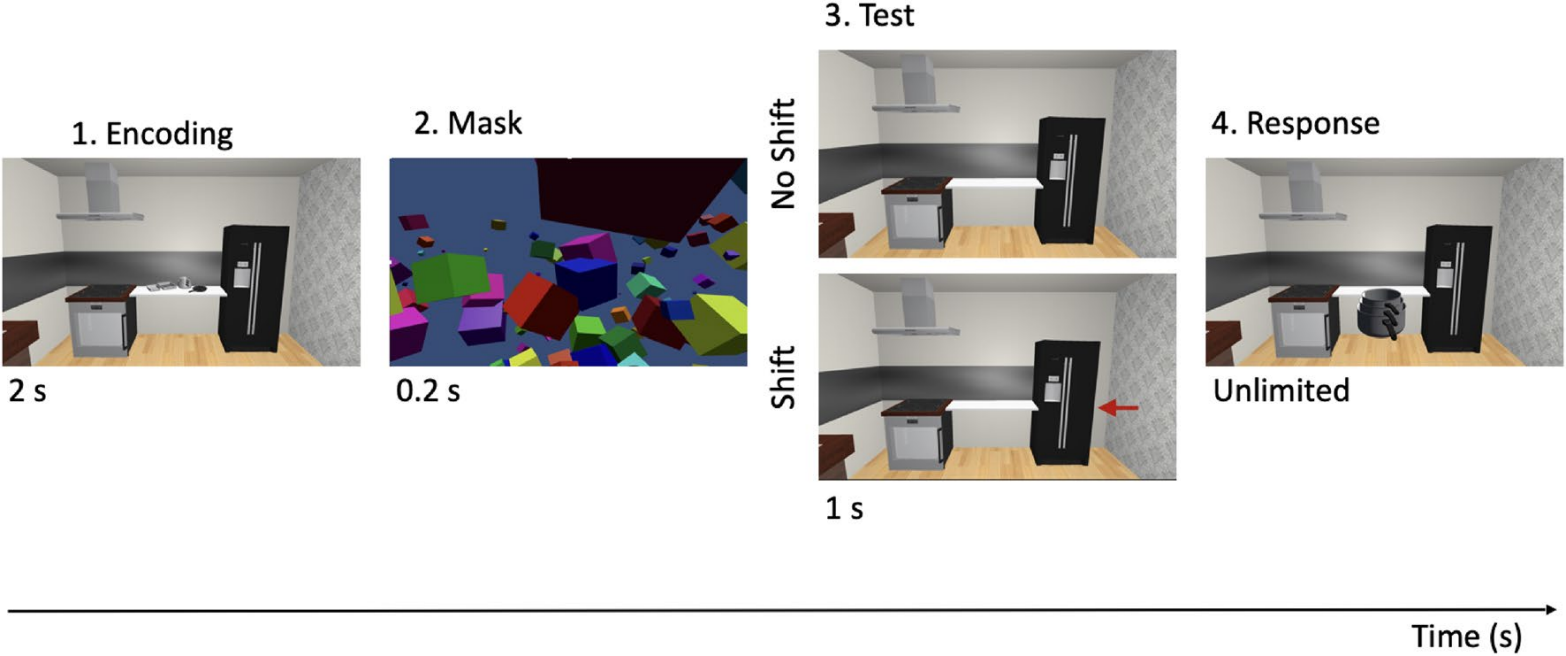
Trial sequence. In a given trial, there are four epochs: (1) Encoding, where participants view the scene with three local objects presented on a shelf extending between two anchors for 2 s; (2) Mask, during which a 3D cuboid mask is presented for 0.2 s; (3) Test, where the scene is presented again with two notable change—there are no local objects present and the anchors are presented in their original locations (No Shift condition) or one of the anchors is shifted 0.075 m leftward or rightward of its original position (Shift condition) (1 s); and (4) Response, which is signalled by a floating local object target that participants must reach out and grab with the controller and walk to place back on the shelf in its remembered location (unlimited duration to complete this step). Of interest to our task were the Encoding, Test, and Response periods. Eye movements were recorded throughout the entirety of a trial, with placement behaviour recorded only at the end of the Response phase.

Each trial was divided into three main events: (1) Encoding + Mask, (2) Test, and (3) Response (Fig. 2). The initial, Encoding phase involved the presentation of the scene (two anchors connected by a shelf, onto which three local objects were presented) for 2 s. This was followed by a 0.2 s three-dimensional (3D) object mask. In the following Test phase the scene was presented again (1 s) with two noteworthy changes: (1) no local objects were present and (2) one of the anchors was either presented in the original location as in Encoding (No shift condition) or shifted leftward or rightward (by 0.075 m; Shift condition). The shift size was determined from a previous pilot experiment and chosen such that participants showed an effect of the shift direction, but that the shift was imperceptible to them. During the last Response phase, one of the three local objects (target) appeared floating approximately 0.3 m in front of the participant at near eye level. They were instructed to grab the target with the controller and then, walk over to the shelf to place it in its remembered location (without having to pay attention to the exact orientation around the vertical axis of the object). They, then, pressed the controller button to indicate the correct placement of the target and to end the trial. This was repeated for each of the three local objects across separate trials, so that each local object became a target once.

There were five trial versions per local object—anchor pairing (one for the No shift condition, and one shift condition (leftward and rightward) for each of the two anchors). There were two sets of three local objects (congruent, incongruent) tested per each of the two anchors for each of the four scenes. In total, 120 trials were split into two blocks to make the total block time manageable for participants. To ensure power and avoid response loss, we repeated both blocks for a total of four blocks, resulting in two full repetitions of all possible combinations tested (i.e., 240 trials in total).

At the end of each experimental session, participants were then asked to fill out a questionnaire, where they were shown each pairing of a local object and anchor that was tested in the experiment. They were asked to rate the strength of the association of the two objects, until all 48 pairings were tested. We used these association strengths as a way of confirming our congruency manipulation of phrasal-level semantics that we tested based on common sense (see Supplementary Materials for additional information). Each experimental session took approximately one hour.

### Placement behaviour data

During the Response phase of every trial, when prompted with the appearance of the target object, participants grabbed the object and placed it on the shelf where they remembered it to have been presented initially. Given that participants were asked to place the object on the shelf and then end the trial when they were content with the indicated object position, we recorded the coordinates of the object in its last position prior to trial end (i.e., at the time of the press of the controller button). Offline, we calculated the horizontal error (X-coordinate) as the difference (in cm) between the centre of the local object in the Response and the Encoding phases. Negative errors were deemed to be leftward shifts, whereas positive errors were taken to indicate rightward shifts of the object, relative to its initial object position. We excluded any errors that exceeded 3 standard deviations (SDs)^13^. On this basis, we included 97.8% (6924 trials out of a total 7080 trials) for further analysis, to which additional exclusion criteria were applied (see following section).

### Eye movement data

In addition to placement responses, we collected raw eye movement data using the Tobii-implemented Vive Pro Eye eye-tracking system^27^. First, we included eye-tracking data only for trials that were analyzed in the placement error analysis. Based on this, we included in eye movement analysis the previous 97.8% of the 7080 total trials recorded in the eye movement analysis. We, then, applied the following exclusion trial- and block-specific criteria. Within a single trial, if more than 20% of the recorded data was found to be missing (due to signal dropout), that entire trial was removed from further analysis. For blocks of eye-tracking data, an entire block was removed from additional analysis if more than 50% of trials were excluded, and if more than 50% of all blocks (four) were missing data, that participant’s data was removed from the analysis altogether. On these bases, we excluded 1 entire block from 1 participant (block criterion 1) and two participants’ datasets (block criterion 2). We performed preprocessing in Python (v3.10) to eliminate these artifacts and outliers, and excluded 4.1% of the total data (i.e., 293 trials; 133 for the Congruent condition, 115 for the Incongruent condition, and 45 for the No Shift condition; by Shift condition, 119 Left shifts (95.8% included), 129 Right shifts (95.6% included), and 45 No Shifts (96.8% included); the exclusion of these data was applied to both eye movement and placement analyses).

### Analysis

#### Placement behaviour data/statistics

To test our specific hypotheses for the effect of (1) Scene-level Semantics and (2) Phrasal-level Semantics, we used a linear mixed modelling approach. We tested models that included anchor shift direction (left, none, right) and phrasal-level semantics (congruent, incongruent) as fixed factors, separately. In each of our tests, we pooled across congruency to test for Scene-level Semantics and across left and right shifts to test for Phrasal-level Semantics (separated according to congruency condition). Random factors that were also tested included: scene (kitchen, bathroom), local target, arrangement, and participant. We chose the final models according to the following criteria: (1) the model should have the lowest Akaike Information Criterion (AIC) score, and (2) the model with the fewest, relevant factors that could best explain the variability in the data^30^. For the Scene-level Semantics hypothesis, we focused only on shift as a fixed factor. For the Phrasal-level Semantics hypothesis, we excluded the No shift trials (no congruency) and tested shift and congruency as fixed factors. Additional post hoc paired t-tests were performed for significant factors that were included in the final model^31^. Any factors that were included in the final model with multiple levels (> 2) were further analyzed in JASP (v0.14.1). A priori hypotheses were tested with additional post-hoc repeated-measures t-tests and Bonferroni corrected, where appropriate, to avoid inflation due to multiple comparisons. Subscripts for comparisons are added to statistical values (*t*-values, *p*-values, and Cohen’s ds) to ensure clarity.

#### Eye movement data/statistics

To determine eye-movement-related effects for Scene-level Semantics and Phrasal-level Semantics, we used four types of behavioural assessment: (1) fixation frequencies, (2) dwell time, (3) fixation distributions, and (4) scanpaths. To determine the first two measures, we extracted pertinent eye movement metrics related to the fixation locations within the virtual environment using a fixation classification package in Python developed by with the following parameters (minimum fixation duration: 0.1 s, maximum angle: 1.62°, minimum frequency: 10 Hz)^32^. Using this approach, we determined (1) the number of fixations produced within each of our three key trial events (Encoding, Test, and Response phases), (2) which objects were fixated during each of the noted fixations, and (3) the time spent looking at each of the respective objects (i.e., mean dwell time, provided in percentage of total epoch time). We averaged across trial types to acquire mean fixation frequencies and dwell times for our shift directions and congruency conditions separately. Given that we have equal number of data points for our mean frequencies and dwell times (which was not the case for our placement error analysis, given that we used all remaining trials for our modelling), we applied repeated-measures ANOVAs (RM-ANOVAs) separately. To these extracted values, we applied statistical analysis in JASP (v0.14.1) for both inferential and descriptive statistics, and visualization tools to graphically represent our findings. To identify gaze behaviour patterns, we generated heatmaps and scanpath gaze plots using custom plot functions in Python (v3.10). For heatmap (fixation distribution visualization) generation, we determined the number of fixations for each pixel in a 2D rendering of the 3D virtual scene for a given scene, arrangement, and target. This was averaged across participants for a particular arrangement (i.e., n = 10; see details related to arrangements in the *Participants* section). These were produced separately for each of the three trial events. Lastly, scanpaths were created using the same 2D renderings mentioned previously; however, these were only produced for fixations that occurred during the Encoding period to determine any potential future influence on spatial coding. The scanpaths plot each fixation as a dot with colours that reflect the order in which they were produced, connected by lines to also highlight the overall trajectory of gaze.

## Results

Given the role that scene semantics has in shaping how we perceive and retain information about our surroundings, for visual search and memory^18,22,33,34^, our study aimed to examine how scene-level and phrasal-level semantics can also shape the spatial relationships that we establish for the use of executing impending goal-directed actions. We tested two specific hypotheses: (1) Scene-level Semantics: shifting an anchor will induce placement errors for local object targets in the direction of the shift (e.g., a leftward anchor shift will induce a leftward placement error in a local object) and (2) Phrasal-level Semantics: shifting an anchor that is semantically congruent with the local object target will induce larger placement errors than for semantically incongruent targets (e.g., a larger placement error will be observed for the yogurt when the fridge is shifted, as compared with a shift of the stove). We also investigated how gaze behaviour is related to the placement behaviour in these memory-guided actions in naturalistic virtual environments by analyzing fixation frequencies, durations, and scanpaths.

### Hypothesis I

**Scene-level semantics influence spatial encoding of local object targets**

**Figure 3:**
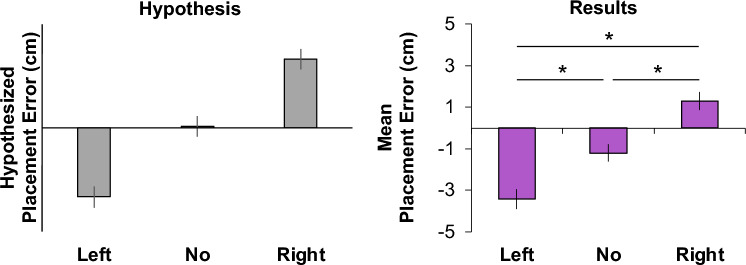
Scene-level semantics hypothesis (left) and placement error results (right). The bar graph on the left depicts the hypothesized placement error (cm) as a function of the Shift (left and right) and No Shift conditions. Negative placement errors suggest a leftward placement error (expected for the Left Shift condition), whereas positive placement errors are indicative of a rightward placement error (expected for the Right Shift condition). The bar graph on the right shows the mean placement errors (cm) for each of the three shift conditions (error bars represent the standard error of the mean, SEM). Asterisks represent statistically significant differences (*p* < 0.05).

We found that the best linear mixed model included Shift as a fixed factor and Participants as a random factor (intercepts only). This suggests that the placement behaviour observed for remembered local object target positions was influenced by and consistent with the direction of the anchor shift (Fig. 3). When we performed additional Bonferroni-corrected post-hoc repeated measures t-tests, we found all three comparisons between the shift and non-shift conditions to be statistically significant (t(29)_Left-None_ =  − 13.46, *p*_Left-None_ = 5.34 × 10^ − 14, Cohen’s d_Left-None_ =  − 2.457; t(29)_Right-None_ = 14.47, *p*_Right-None_ = 8.50 × 10^ − 15, Cohen’s d_Right-None_ = 2.641; t(29)_Left–Right_ =  − 19.75, p_Left-Right_ = 2.32 × 10^ − 18, Cohen’s d_Left-Right_ =  − 3.605). These findings suggest that anchors, though not relevant to the instructed task, influence the positioning of the remembered local objects.

### Hypothesis II

**Phrasal-level semantics influence spatial encoding of local object targets**

**Figure 4:**
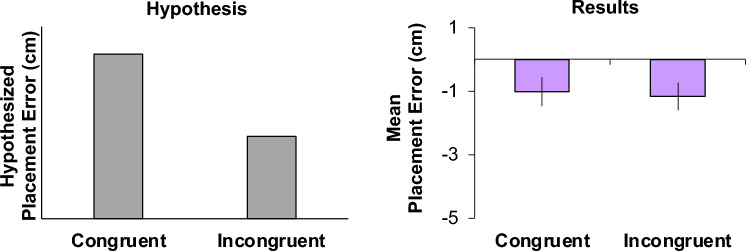
Phrasal-level semantics hypothesis for placement behaviour (right) and placement error results (right). In the bar graph on the left is the hypothesized placement error (cm) as a function of phrasal-level semantics (Congruent condition, where the local object is semantically congruent with the shifted anchor, and Incongruent condition, where the local and anchor pairs are semantically incongruent). We hypothesized that the placement error would be larger, regardless of direction (positive or negative placement errors), for the Congruent compared with Incongruent condition. If there were an effect, there would be a significant difference in placement error between the two conditions. In the bar graph on the right, depicted are the mean placement errors (error bars represent SEMs) for the Congruent and Incongruent conditions.

In contrast to the hypothesis, we found that there was no effect of Congruency on the placement errors (F(1, 5471) = 1.1215, *p* = 0.2896). This suggests that, at least in this experiment, there was no phrasal semantic influence of anchors on the spatial coding of local objects (Fig. 4). Ultimately, anchors influence the spatial encoding of local objects, independent of their phrasal-level semantics.

### Gaze is directed exclusively to local objects, irrespective of phrasal-level semantics

We also aimed to understand the role that eye movements have in supporting goal-directed memory-guided actions. We quantified eye movement behaviour in fixation frequencies and dwell time during the Encoding phase. We assessed the results in two steps: (1) We determined how these two parameters were influenced by local objects versus anchors and (2) when fixations landed on anchors, we investigated if they showed an influence of phrasal-level semantics.

**Figure 5:**
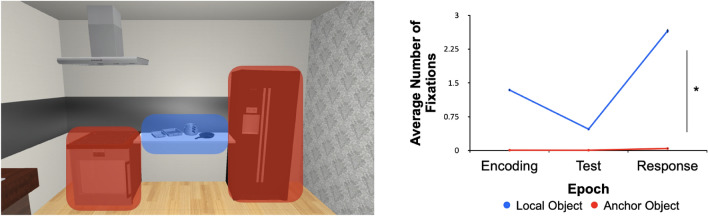
Region-of-interest (ROI) results for fixation frequencies. On the left is an example kitchen scene with two types of regions indicated (red objects reflect the anchor region, AR, whereas blue objects reflect the local object region, LOR). Mean fixation counts (error bars depict SEMs) are reported in the graph on the right as a function of our three epochs of interest. Overall, there is a clear difference between the fixations toward local objects (blue) and anchors (red).

**Figure 6:**
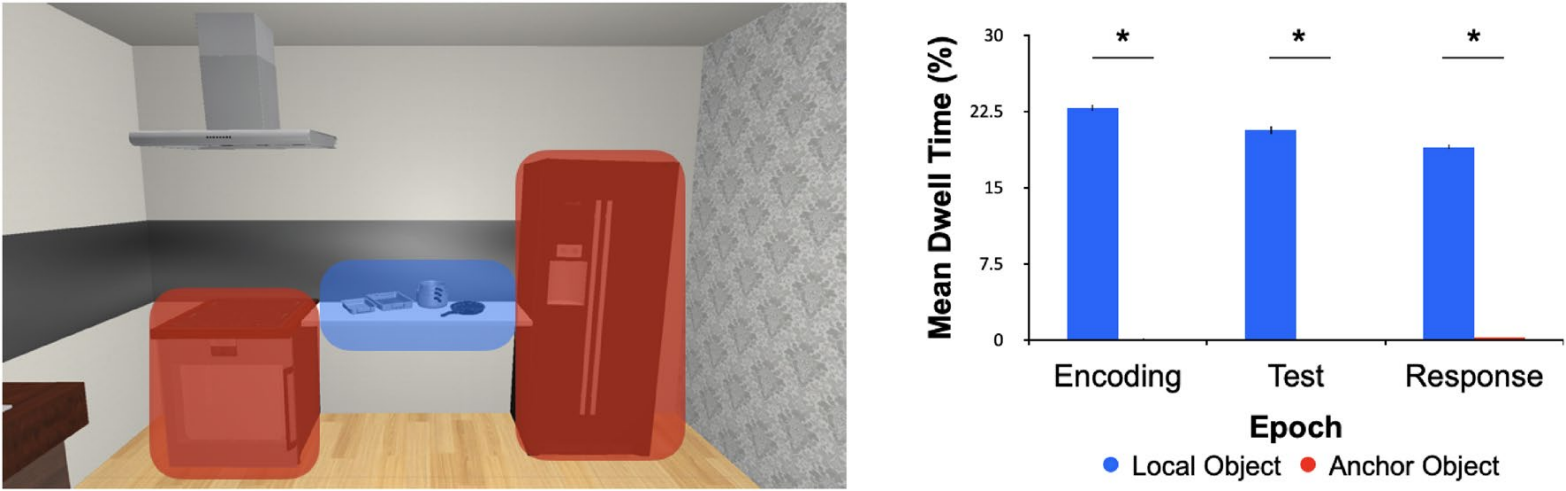
Mean dwell time results for region-of-interest analysis. The example scene on the left depicts the two regions analyzed, namely the anchor (red) and local object (blue) regions. The dwell time (percentage of each epoch dedicated to looking at the local or anchor regions) results are plotted in the graph on the right. The mean dwell times for local (blue) and anchor (red) objects are plotted (error bars represent SEMs) for each of the three trial epochs. There are significant (asterisk, *p* < 0.05) differences in dwell time between the two regions.

First, we took each scene and created two main regions-of-interest (ROIs)—namely, the Local Object Region (LOR) and the Anchor Region (AR). We determined fixation frequencies and dwell times for each of our ROIs for any fixations that landed on any of the local objects (i.e., LOR) or anchors (i.e., AR). For fixation frequencies, there was a significant effect of object type (F(1,29) = 4.28; *p* = 0.048; η^2^ = 0.076), trial epoch (F(1,29) = 4.25; *p* = 0.048; η^2^ = 0.028), and their interaction (F_1,29_ = 4.24; *p* = 0.048; η^2^ = 0.024). We found that fixation frequencies were disproportionately greater for local objects than for anchors (Fig. 5). This same pattern was also observed for dwell times (main effect of object type: F_1,29_ = 4.23, *p* = 0.048, η^2^ = 0.126; no main effect of epoch: F_1,29_ = 3.72, *p* = 0.058, eta-squared = 0.001; and no interaction: F_1,29_ = 3.80, *p* = 0.057, η^2^ = 0.001) (Fig. 6). This shows that participants primarily fixated the task-relevant local objects without the need to look at the task-irrelevant anchors. However, incidental (but nearly zero) fixations also occurred on anchors, especially during the Response phase (Fig. 6).

**Figure 7:**
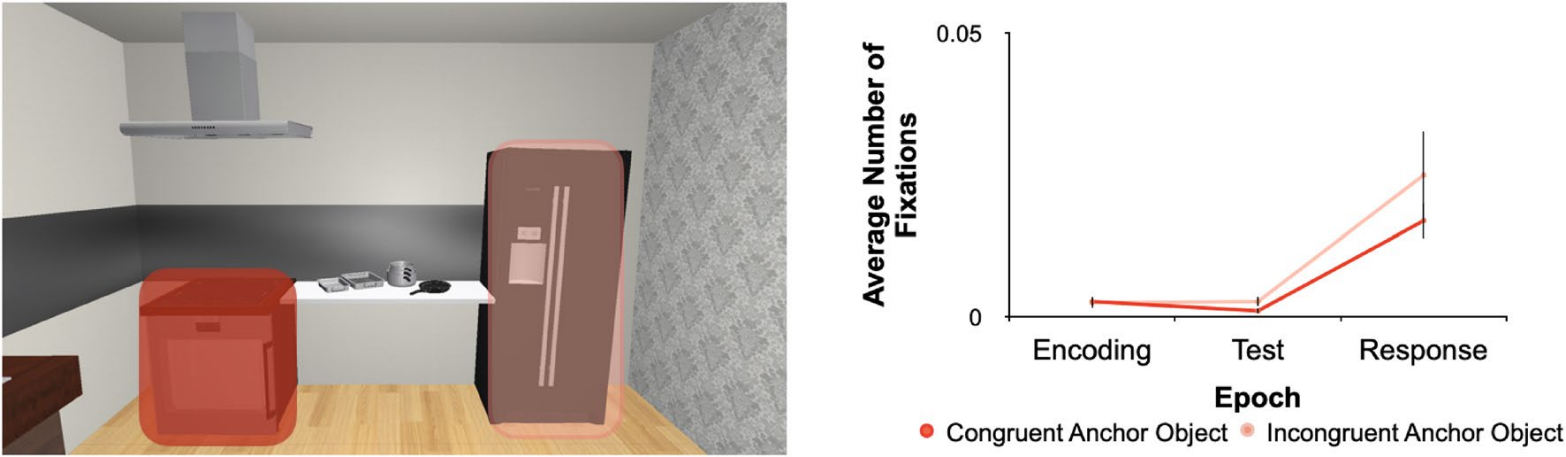
Region-of-interest analysis results for fixation frequencies for semantic context. In the same scene on the left as in Fig. 5, we segmented the anchor regions further for the phrasal-level semantic Congruent (cherry red) and Incongruent (peach) conditions. In the graph on the right the mean number of fixations (error bars represent SEMs) as a function of the phrasal-level semantic conditions is presented. There is no significant difference between the conditions.

**Figure 8:**
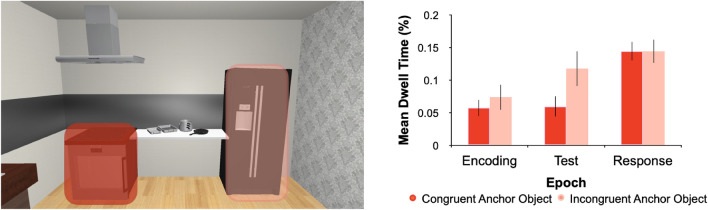
Region-of-interest results for dwell time. In the example scene on the left are depicted regions where dwell time was analyzed for congruent (cherry red) and incongruent (salmon) anchors. In the graph on the right are mean dwell times (percentage of each epoch spent fixating on a given anchor; error bars are SEMs) across the three trial epochs for the Congruent and Incongruent anchors. There are no significant effects of scene semantics for dwell time.

In a second step, we looked at whether fixations that landed on anchors were affected by the phrasal-level semantics. For each trial during Encoding, we assessed whether any of the fixations that landed on an anchor was directed toward the congruent or incongruent anchor. We neither found an effect of phrasal-level semantics (F_1,29_ = 0.93, *p* = 0.34, η^2^ = 3.45 × 10^ − 4), trial epoch (F_1,29_ = 3.91, *p* = 0.057, η^2^ = 0.115), nor an interaction (F_1,29_ = 0.24, *p* = 0.66, η^2^ = 1.50 × 10^ − 4). Fixation frequencies were equivalent across phrasal-level semantics, i.e., fixations were similar for Congruent and Incongruent conditions (Fig. 7). Dwell time assessments showed a similar pattern (main effect of semantics: F_1,29_ = 0.019, *p* = 0.89, η^2^ = 1.15 × 10^-5; no main effect of epoch: F_1,29_ = 3.78, *p* = 0.059, η^2^ = 0.093; and no interaction thereof: F_1,29_ = 3.44, *p* = 0.068, η^2^ = 0.019). In terms of phrasal-level semantics, fixation durations were not significantly different between the Congruent and Incongruent conditions (Fig. 8). Taken together, these two metrics suggest that eye movements produced during the Encoding phase of a trial were consistent with (and predictive of) placement behaviour, in that both behavioural measures showed no influence of phrasal-level semantics.

**Figure 9:**
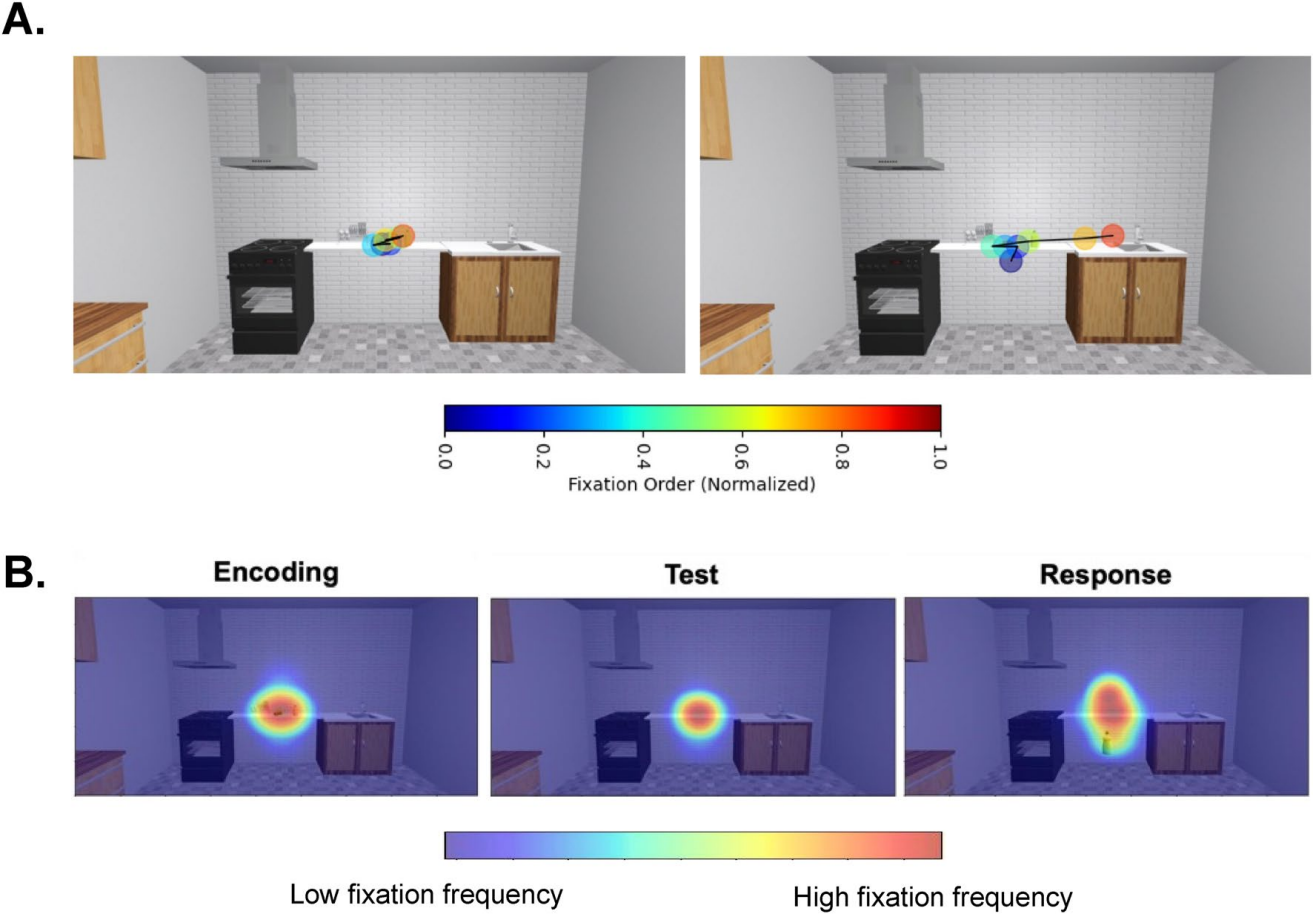
Depiction of scanpaths (top row) and heatmaps of fixations across a trial (bottomrow). (**A**) A typical scanpath of the Encoding phase which shows central tendency bias (left panel), where fixations are drawn to the centre of the scene and where the task-relevant, local objects are found along the shelf. A scanpath during Encoding is shown (right panel), where the last couple of fixations land on the anchors. These situations are infrequent. The colour bar represents the normalized fixation order, where cooler colours represent early fixations and warmer ones depict later fixations. (**B**) In the Encoding phase, fixations are centred on the shelf and spread across the local objects. During the Test phase, the absence of the local objects is reflected in the tightening of the spread of fixations along the shelf. Lastly, the Response phase shows a spread of fixations across where the target object appears and the shelf where the object must be re-placed. This is accompanied by a spread of activation across these two key regions during this last trial event. Overall, these heatmaps show event-related activity for this naturalistic VR task.

We further visually inspected gaze patterns via scanpaths during the Encoding phase as well as averaged fixation distributions (heatmaps) for each of the three main trial events. Scanpaths confirm a tendency to maintain fixation on the local object region, given their importance for the task. We determined that, when gaze was directed toward an anchor, it would often occur within the last two fixations of all fixations during Encoding (Fig. 9). When we averaged fixations across the three trial epochs, we observed a similar pattern for the scanpaths during Encoding – a tendency to produce fixations on local objects that extends across the local object area (from end to end) (Fig. 9). When the local objects were no longer present during the Test phase, fixations became more focal and centralized. Lastly, during the Response phase, fixations landed on the (1) local target object floating in front of the participant and (2) the shelf area (for the upcoming re-placement) with greater dispersion of gaze (Fig. 9). Gaze behaviour also reflects event- and task-related activity, as demonstrated by the change in fixation spread across the three events. Overall, gaze behaviour supports our findings on the placement behaviour, showing no effect of phrasal-level semantics for memory-guided actions.

## Discussion

The placement of objects in scenes is governed by the nature of the actions we perform therein. Large objects act as anchors for interaction with objects within our environments^17,22,35,36^. Given the role that anchors play in helping us to predict the locations of local objects that we frequently interact with, we aimed to understand what role they play in the spatial coding for memory-guided actions. By modulating different aspects of scene grammar, we tested the degree to which scene-level and phrasal-level semantics influence the spatial coding of local objects within two scene types that evoke strong anchor-local object relationships. We hypothesized that we would find an influence of the shifted anchors on the remembered position of local object targets (Scene-level Semantics hypothesis). Based on previous work^16^, we expected that encoding of local object positions would be influenced by the semantic relationship between local objects and anchors (Phrasal-level Semantics hypothesis). In our investigations here, we found that anchors *do* influence the allocentric spatial coding of local objects, but in a manner that is *irrespective* of their phrasal semantic relationship.

### Spatial coding and task (ir)relevant objects

Previous investigations demonstrate that task relevance can play an important role in allocentric coding of objects in naturalistic scenes^13,14^. In the study by Klinghammer and colleagues^13^, anchors (i.e., a breakfast table) were spatially stable and investigations focused on local—local object interactions. The authors found that local objects that were relevant to the task were influential for the spatial coding of a local object target, whereas task-irrelevant objects were ineffective. In the present study, we never instructed participants to pay attention to anything other than the local targets. We also had participants remain stationary until the point of Response, when they were required to walk and place the object. Under such circumstances, the spatial coding effect may have been mitigated by confounding optic flow signals during walking^37^; however, participants initially encoded object (local and anchor) locations from a stationary vantage point during initial scene presentation, as well as during the Test phase, where an anchor shift could have occurred. Thus, we suspect that optic flow signals were less relevant here, but further investigation of the influence of optic flow on spatial coding in real-world scenes would be insightful. Nevertheless, participants seemed to be influenced by anchors, despite not being relevant to fulfill the requirements of the task. This speaks to the idea that anchors possess a privileged role for scene perception and actions that we perform therein^22,35,36^. When participants are asked to create virtual scenes from scratch, they create a layout by first placing anchors and subsequently arranging local objects accordingly^22^, which influences their search and recall behaviour as well. It has also been shown that gaze can fall onto task-relevant and -irrelevant stimuli, the latter of which still play a role in improving an overall spatial layout and memory that can ultimately improve task-relevant performance^38–40^. Thus, it is rather surprising that participants would not implicitly relate local object positions to the seemingly irrelevant anchors in the scene, given the supportive role that task-irrelevant anchors might play. The influence we observed here might also be driven by the fact that anchors were not far in the periphery when participants were fixating on the local objects, making it easy to incorporate these spatial relations into the allocentric representation of local object targets. We kept the distance between anchor and local objects constant here, but it would be of importance to determine at what spatial separation anchors exhibit little-to-no influence for the allocentric coding of local objects for memory-guided actions, much akin to how reachable versus unreachable space for perception has been previously defined^23,41,42^. Lastly, in putting into perspective the task-relevant results for spatial coding of objects found by Klinghammer et al.^13^, Fiehler and Karimpur^3^ put forth the hypothesis that allocentric coding might be associated with explicit task instruction. Given that we provided no explicit instructions about the use of anchors in guiding remembered local object positions, it is possible that (explicitly indicated) task relevance might also have different interactions along the scene grammar hierarchy^5,17^.

### When phrasal-level semantics for spatial coding do not apply

While the majority of investigations into spatial coding have largely relied on abstract stimuli and scenes^1,4,8–10^, it has been shown with the use of carefully created naturalistic stimuli how other, increasingly complex, cognitive factors can serve to influence allocentric coding^5,11–14,16^. The only other study that investigated the influence of semantics on allocentric coding (also in VR) identified that the semantic interplay between target and surrounding local objects influences how target positions are encoded and later utilized for goal-directed actions^16^. Based on previous findings^16^, we expected to find a difference in behaviour as a result of the semantic relationship at the phrase level between anchor and local objects (i.e., larger placement errors for anchors semantically congruent with the local object target). This hierarchical organization also applies to the semantics of actions^43^. When participants were tasked with matching a word to a photograph of a tool, performance was poorer for cases where objects belonging to the same semantic action category were presented simultaneously than when objects from different semantic action categories were shown^34,43^. While there is less influence in the placement error on the semantically congruent condition in our task (consistent with the previous idea), it is not significantly different from the incongruent condition. In a separate association rating task following the experiment we were able to use and confirm our chosen anchor—local objects pairings for semantic categorization. Therefore, it is not a likely contributing factor to our findings here. The role of phrasal-level semantics (as comprised within scene grammar) was stronger and more important in previous tasks, where participants had to actively create scenes and then, perform recall tasks^22^ or perform visual search within naturalistic virtual scenes^35^. It may suggest that phrasal-level semantics drive behaviour in a task-dependent manner. Based on the aforementioned findings, we expected that the semantic link between local and anchor objects would translate to other types of tasks that include locating objects as well, such as the memory-guided placement task^13^ we used here. However, this was not the case. Our findings instead provide evidence that in this set-up, anchors could easily be ignored (as seen in close to zero fixations) and thus it is not the mere need to remember the location of objects that activates phrase-sensitive representations. There is obviously still a need to better understand at what level of the scene grammar and action hierarchies semantics can influence goal-directed actions for spatial coding.

### The contribution of eye movements to spatial coding in naturalistic scenes

Oculomotor behaviour can provide insight into different strategies of spatial encoding of objects for memory-guided actions. For example, when participants were instructed about task-relevant objects, there was a clear demonstration that oculomotor behaviour mimicked placement behaviour for spatial coding, where fixations were clustered on table objects in a task involving table items or on background objects for a task involving background items^13^. When participants’ eye movements were either restricted (fixation condition) or were allowed to move freely (free-viewing condition), it was found that eye movement behaviour influenced the strength of allocentric coding showing larger object shift errors when gaze was free than restricted to central fixation^12^. In the present study, participants could freely move their eyes. Despite this flexibility, most fixations were directed to the area where local objects were presented (Figs. 5 and 6). When we break down these results according to the regions of the scene (i.e., local object regions and anchor regions; Figs. 5 and 6), we found that eye movements were predominantly focused on the local object regions, consistent with the task-relevant region of the scene. The local objects were presented centrally in the scene, with heatmaps showing that it is largely central tendency bias at play, but with additional slight modulation shown in the spread of fixations in an event-related manner. Given how close the anchors were to the local objects, it is also clear why anchors could have affected spatial coding of the local objects. Were participants standing any closer to the local objects, attenuating the visual extent of anchor viewing, it may have been the case that anchors would have had a diminished effect on local object coding.

When we assessed fixation durations and dwell time in our regions-of-interest, we found that participants hardly looked at anchors (Figs. 5 and 6). This is likely a result of the positioning of the participant which was always indicated such that they could see both anchors in their visual periphery when fixating on the local objects. The infrequent gaze on anchors, coupled with the similar eye movement behaviour across semantic categories, during the initial, Encoding event predicted the placement behaviour that we observed during the Response phase. While we chose local objects that we deemed (and confirmed) to be congruent or incongruent with the anchors, all local objects were scene congruent. Future studies could look at whether scene-level semantics play a role in spatial coding by presenting objects that are either congruent or incongruent to the scene as a whole.

## Conclusion

In this study, we set out to better understand the interplay between scene-level and phrasal-level semantics with allocentric spatial coding for memory-guided actions. Placement behaviour in this virtual reality task indicates that anchors are used to guide allocentric coding of local objects. However, this occurs in a manner that is phrase-insensitive in that the semantic nature of the local-anchor object relationship is irrelevant. Overall, anchors influence allocentric spatial coding of local objects in our complex surroundings for memory-guided actions, regardless of their phrasal associations.

### Supplementary Information

Supplementary Information.

## Data Availability

All data (placement results, eye-tracking data, and association strengths) are available on OSF (https://osf.io/zf2e4/?view_only=8733575af09f4eec8ed60af9da9f008a).
