# Supplementary material for: Scene semantics affects allocentric spatial coding for action in naturalistic (virtual) environments
Source: Sci Rep. 2024 Jul 5;14:15549. doi: 10.1038/s41598-024-66428-9 (PMC11226608; doi:10.1038/s41598-024-66428-9)
Supplement: Supplementary file 1 — Supplementary Information. [file 41598_2024_66428_MOESM1_ESM.docx]

**Supplementary Material**

**Title: Scene semantics affects allocentric spatial coding for action in naturalistic (virtual) environments**

**Author(s) and Affiliations:** Bianca R. Baltaretu, Immo Schuetz, Melissa L.-H. Võ, & Katja Fiehler

**Supplementary Methods**

**Analysis**

**Association Strengths / Statistics.**

We applied a repeated-measures analysis-of-variance (RM-ANOVA) to the association strengths, averaged across the local-anchor object combinations, for each of the four scenes and phrasal-level semantics (congruent, incongruent) that were tested. We were interested in determining whether there were 1) differences between the congruent and incongruent conditions ratings (to validate the preselected local-anchor pairings) and 2) scene-related differences in the pairings tested. We, then, applied additional post-hoc paired t-tests (with Bonferroni correction, applied wherever appropriate) to identify any scene type (kitchen vs. bathroom) and phrasal-level semantic (congruent vs. incongruent) effects (JASP, v0.14.1).

**Supplementary Results**

**Association strengths confirm semantic combinations.**

We tested whether our common-sense combinations between anchor and local objects were deemed congruent and incongruent by our participants. After applying an RM-ANOVA to the ratings, we found that there was a significant effect of phrasal-level semantics (F_1,1_= 178.07, p= 6.58 x 10^-14^, η^2^= 0.72), a significant effect of scene (F_1,3_= 3.42, p= 0.021, η^2^= 0.01), and a significant interaction thereof (F_1,3_= 9.08, p= 2.70 x 10^-5^, η^2^= 0.016). From further post hoc paired t-tests, we found that there was a significant difference between B01 and K02 (cf. Fig. 1), in terms of association strengths (t_1,3_= 3.09, p= 0.016, Cohen’s d= 0.56). Lastly, all combinations were tested and several significant differences were found (see Supplementary Table 1), with noteworthy differences between B01 (Congruent) and B01 (Incongruent) (t_1,3_= 11.287, p= 1.14 x 10^-14^), B02 (Congruent) and B02 (Incongruent) (t_1,3_= 9.026, p= 7.02 x 10^-12^), K01 (Congruent) and K01 (Incongruent) (t_1,3_= 13.409, p= 9.065 x 10^-18^), and K02 (Congruent) and K02 (Incongruent) (t_1,3_= 10.655, p= 8.148 x 10^-15^). Overall, these results suggest that there were appropriate combinations between anchor and local objects to test the Phrasal-level Semantics Hypothesis.

**Supplementary Table 1**. Post hoc paired t-tests that evaluate the differences between the two phrasal-level semantics conditions (Congruent, Incongruent) and the four scenes (B01, B02, K01, and K02), with standard error (SE), t-values, and Bonferroni-corrected p-values provided.


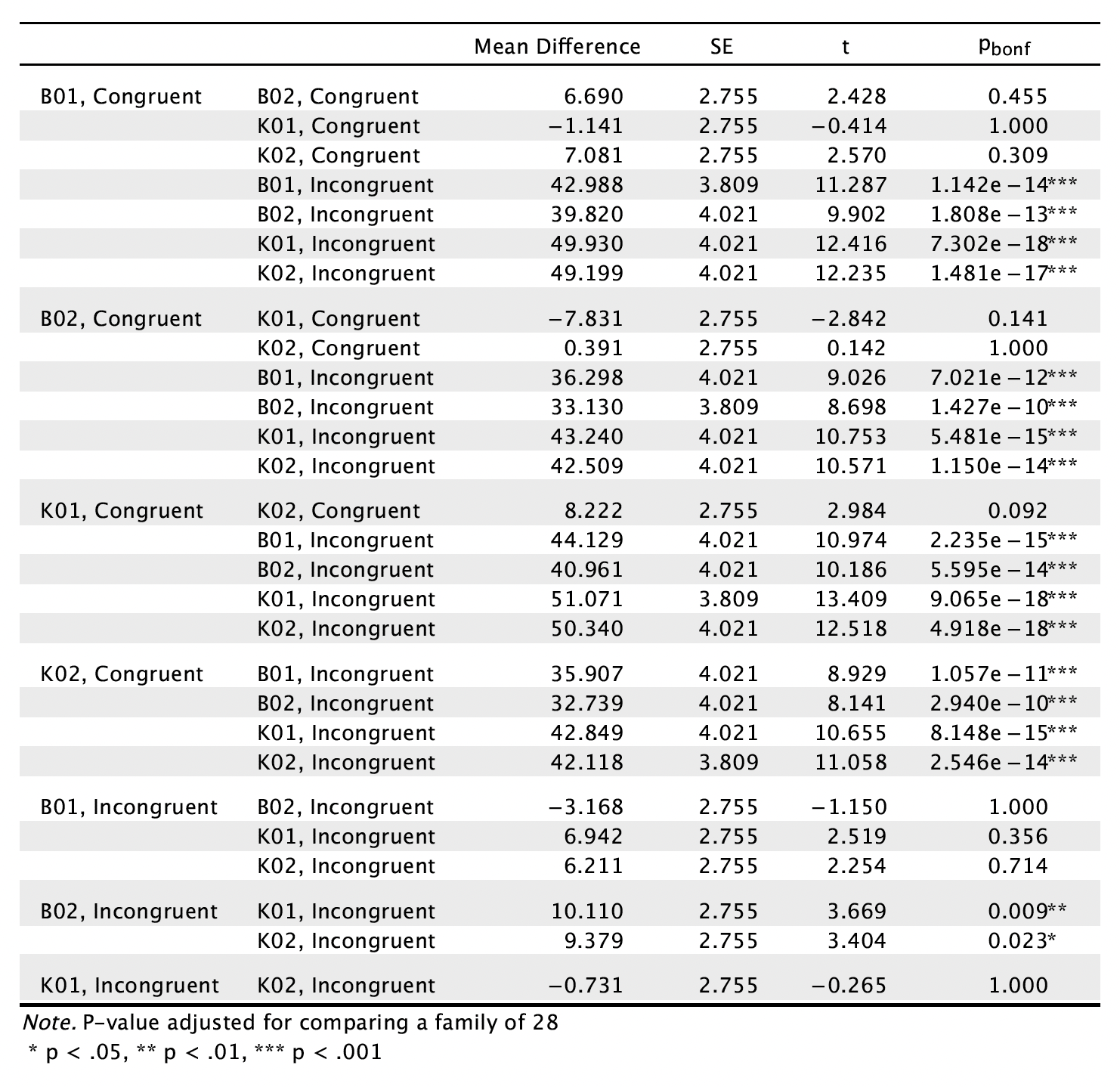


**Linear Mixed Model Comparisons for Scene - and Phrasal-level Semantics**

In testing for Scene-level semantics, we included Shift as a fixed factor (Left, Right, and No shift), which are also coded for Congruency (coded for shift conditions only). We only fit Model 1 (see Supplementary Table 2) to the data in order to test for effects of anchor shift on the placement errors. The results indicate an effect of Shift (F(2, 6869) = 512.36, p = <2.2 x 10^-16^).

In testing for Phrasal-level semantics, we included Shift and Congruency as fixed factors in our linear mixed model. We found a significant effect of Shift (only Left and Right shifts included here; F(1, 5471) = 1018.0154, p = < 2.0 x 10^-16^). We did not find a main effect of Congruency (F(1, 5471) = 1.1215, p = 0.2896), nor an interaction between the two factors (F(1, 5471) = 0.0059, p = 0.9386). These findings suggest that there is no influence of phrasal-level semantics on placement errors.

**Supplementary Table 2.** Models tested for Scene- and Phrasal-level Semantics, with the fixed (Shift, Congruency) and random factors (Target, Participant), and the related AIC value (data are directional placement errors; No shift condition trials are excluded in Model 2).

| **Model** | **Factors** | **AIC** |
| --- | --- | --- |
| 1 | Shift + (1\|Target) + (1\|Participant) | -20262.31 |
| 2 | Shift + Congruency + Shift*Congruency + (1\|Target) + (1\|Participant) | -16104.06 |

**Pilot Experiment**

To determine the appropriate shift size for the experiment detailed here, we had 14 participants who completed a similar memory-guided placement task. Participants had to re-position a local target object onto an anchor object that either 1) was presented in its original position or 2) was shifted (either leftward or rightward). We tested several shift sizes, from 5 to 25 cm in 5 cm increments, in both directions (leftward and rightward). At the end of each trial, participants were prompted to make a binary response about whether they noticed any change in the scene (i.e., a way for us to assess if the shift became perceptible to participants and if so, for which shift magnitude).

In a typical trial, participants started off by viewing Kitchen 1 (Fig. 1) only, where one of several local objects was presented on neutral table or on the stove (anchor objects). A mask was then presented for 200 ms. After this, the scene was presented again with the local object missing and with anchor shift implemented (Shift) or not (No shift). After this, the local target object appeared in front of the participant, who then had to grab and re-place the object in its remembered position.

As in this study, we determined placement errors for each of the No shift and Shift conditions, for each of the anchors. We also determined the relationship between shift size and when participants noticed a change by plotting psychometric functions. On these bases, we determined that participants showed an effect of shift from 5 cm (either leftward or rightward) and indicated consistently awareness of the shift from 10 cm and upwards. Taken together, we deemed that 7.5 cm would be an appropriate middle ground, as it was large enough to exhibit an influence on spatial coding and was simultaneously small enough to go unnoticed by participants.
